# Analysis of the cellular centrosome in fine-needle aspirations of the breast

**DOI:** 10.1186/bcr1752

**Published:** 2007-07-29

**Authors:** Hui-qin Guo, Meixia Gao, Jinfang Ma, Ting Xiao, Lin-lin Zhao, Yanning Gao, Qin-jing Pan

**Affiliations:** 1 Department of Pathology, Cancer Institute/Hospital, Peking Union Medical College & Chinese Academy of Medical Sciences, Beijing 100021, PR China; 2 Department of Etiology and Carcinogenesis, Cancer Institute/Hospital, Peking Union Medical College & Chinese Academy of Medical Sciences, Beijing 100021, PR China

## Abstract

**Background:**

The purpose of the present investigation is to determine whether centrosome amplifications are present in breast tumor cells, whether there are differences of centrosome amplification between benign breast lesions and breast carcinomas, and whether centrosomal analysis can be of value in the diagnosis and prognosis of breast carcinoma.

**Methods:**

Using immunofluorescence analysis with an antibody against γ-tubulin, we analyzed centrosome abnormalities in fine-needle aspirations of 100 breast lesions (25 cases with benign lesions and 75 cases with carcinomas).

**Results:**

We found that centrosome amplifications, including numerical centrosome amplification and structural centrosome amplification, were present in most breast tumors. Cells with numerical centrosome amplification were found in 23 of 25 benign lesions, and in all 75 cases of breast carcinomas. Cells with structural centrosome amplification were found in three of 25 benign lesions, and in 69 of 75 breast carcinomas. The breast carcinomas showed a mean percentage of cells with numerical centrosome amplification of 4.86% and a mean percentage of cells with structural centrosome amplification of 3.98%. These percentages were significantly higher than those in benign lesions, with a numerical centrosome amplification of 2.77% and a structural centrosome amplification of 0.10%. Furthermore, the mean percentage of cells with structural centrosome amplification was significantly associated with HER2/neu overexpression (*P *< 0.05) and with negative estrogen receptor status (*P *< 0.05), and had a borderline association with negative progesterone receptor status (*P *= 0.056) in breast carcinomas.

**Conclusion:**

Structural centrosome amplification may bear a close relationship with breast carcinoma and may be a potential biomarker for diagnosis and prognosis of breast carcinoma.

## Introduction

The centrosome consists of a pair of centrioles surrounded by electron-dense pericentriolar material, and represents the microtubule organizing center of interphase and mitotic cells. Because the centrosome plays an important role in the maintenance of cellular polarity and chromosome segregation during mitosis, the characteristic loss of cell polarity and abnormal chromosome number (aneuploidy) commonly seen in human malignant tumors could result from defects in the centrosome [1-3]. To date, centrosome amplifications are found in the vast majority of human malignant tumors, including those of the pancreas, the prostate, the breast, the lung and the colon [4,5]. In a xenograft model of pancreatic cancer, centrosome amplification might cause the tumor to progress to a more advanced stage [6].

In the present study, we analyzed centrosome aberrances in fine-needle aspirates (FNAs) of breast tumors, evaluated the differences of centrosome amplification between benign breast lesions and breast carcinomas, and studied the relationships between centrosome amplification and the diagnosis, as well as the prognosis, of breast carcinoma.

## Materials and methods

### Patient samples

Breast tumors resected from patients in the Cancer Hospital of the Chinese Academy of Medical Sciences (CAMS) from March to September 2006 were aspirated with a 23-gauge needle attached to a 10 ml syringe, and the samples of aspirations were rinsed into a test tube containing 20 ml CytoLyt solution (Cytyc Corporation, Marlborough, MA, USA). The use of human tissue samples and the experimental procedures for this study were reviewed and approved by the ethics committee of the Cancer Institute/Hospital, CAMS.

### Liquid-based preparation

The tubes containing the patient samples were concentrated by centrifugation for 10 minutes. The supernates were poured off and the cell pellets were vortexed to become resuspended. Specimens were added to a PreservCyt solution vial (Cytyc Corporation) and were allowed to stand in the vial for 15 minutes. Vials were then loaded into the ThinPrep 2000 processor (Cytyc Corporation). After the machine was run using sequence 2, the monolayer slides were made. For each case we made two slides, one for cytology diagnosis and another for centrosome labeling.

### Centrosome labeling

The ThinPrep slides were immunostained with an antibody against γ-tubulin, using the following steps. The slides were fixed in methanol at -20°C for 30 minutes and in acetone at -20°C for 6 minutes, were permeabilized in buffer (0.1 M piperazine-N, N'-bis-2-ethanesulfonic acid buffer (pH 6.9), 1 mM ethylene glycol-bis(2-aminoethyl ether)-N,N,N',N'-tetraacetic acid, 4 M glycerol, 0.5% Triton X-100, and 1 mM guanosine triphosphate) for 5 minutes [7], and were immersed in 3% hydrogen peroxide in PBS for 10 minutes to block endogenous peroxidase. The slide was then heated in antigen retrieval solution in a microwaveable pressure cooker for 30 minutes. Blocking solution (10% normal goat serum, 2% BSA in PBS) was applied to the slides for 30 minutes and the slides were incubated with mouse anti-γ-tubulin monoclonal antibody (diluted 1:200 in PBS; Sigma, St Louis, MO, USA) overnight at 32°C. The antibody–antigen complexes were detected by a rhodamine-conjugated antibody after incubation for 30 minutes at 37°C. Between the incubations, the slides were washed extensively with PBS containing 0.1% Tween 20. The slides were finally counterstained with 4',6-diamidino-2-phenylindole (Vector, Burlingame, CA, USA) and examined under a fluorescence microscope (Olympus BX-51; Olympus, Tokyo, Japan). The centrosome images were obtained with the aid of the image analysis system CytoVision^®^2.7 (Applied Imaging, Newcastle, UK).

### Calculation of centrosome amplification

Centrosome images from cells of normal mammary tissues were used as controls. A normal centrosome was detected as one or two regular rounded spots of uniform size and shape [4,8]. If there were any centrosomal changes in number and/or in shape, the centrosome was considered aberrant. The cell was considered a cell with numerical centrosome amplification if it had three or more centrosomes. The cell was considered a cell with structural centrosome amplification if the diameter of its centrosome was greater than twice the diameter of the normal centrosome and/or if the shape of its centrosome became irregular.

We calculated the percentages of the cells with numerical centrosome amplification and of the cells with structural centrosome amplification in one case respectively. The percentage of the cells with centrosome amplification was determined by dividing the number of cells with centrosome amplification by the number of investigated cells. At least 200 cells per slide were examined. All slides were evaluated without knowing the diagnosis of cytology and histology.

### Cytology diagnosis

For cytological diagnosis, the slides were fixed in 95% alcohol, were stained by hematoxylin and eosin, and were evaluated by two cytopathologists in a double-blinded manner. The diagnoses were categorized into three groups: benign, suspicious carcinoma, and carcinoma.

### Statistical analysis

Patient data including the tumor size, the histological category and grades, the lymph-nodal status, the estrogen receptor (ER) status, the progesterone receptor (PR) status, the Her2 status, and DNA ploidy were obtained from the patients' files. The final histology results were used as the golden standard. Correlations of centrosome amplification with histological diagnosis and pathologic prognostic variables (tumor size, histological grades, lymph-nodal status, ER status, PR status, Her2 status, and DNA ploidy) in breast carcinomas were assessed via a *t *test for quantitative data and via the Mann–Whitney U test or the chi-square test for qualitative data. *P *< 0.05 was considered statistically significant. All statistics were calculated with the aid of SPSS, version 12.0 (SPSS, Chicago, IL, USA).

## Results

In total, 100 FNAs of breast lesions were included in our study, including 25 cases of benign lesions, two cases of ductal carcinoma *in situ *and 73 cases of invasive carcinomas characterized by their centrosomal status. The benign lesions consisted of 15 fibroadenomas, five mastopathias, two introductal papillomas, one case of fibrocystic mastopathy, one case of mastitis and one case of fatty necrosis. The invasive carcinomas included 72 cases of invasive ductal carcinomas and one case of invasive lobular carcinoma (Table 1).

**Table 1:** Patient age and histology diagnosis

Age (years)	
Benign tumor	33 (20–50)
Malignant tumor	50 (29–73)
	
Histological type	
Fibroadenoma	15
Mastopathia	5
Introductal papilloma	2
Fibrocystic mastopathy	1
Mastitis	1
Fatty necrosis	1
Ductal carcinoma *in situ*	2
Invasive ductal carcinoma	72
Invasive lobular carcinoma	1

Data presented as the mean (range) or as *n*.

### Centrosome expression

**Figure 1:**
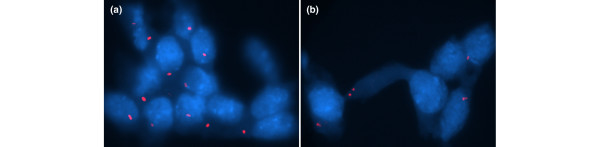
Normal centrosome staining of benign tumors. **(a) **Fibroadenomas and **(b) **mastopathias show one or two rounded spots in uniform size and shape. (b) Also shows centrosomes near the cell membrane.

**Figure 2:**
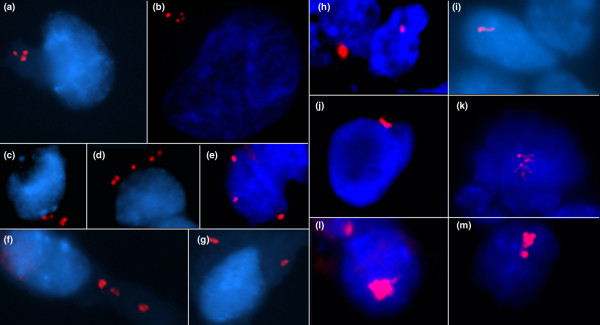
Centrosome amplifications. **(a)–(g) **Centrosomes with numerical amplification: (a), (d), (f) from fibroadenoma, (b) and (e) from carcinoma, and (c) and (g) from mastopathias. **(h)–(m) **Centrosomes with structural amplifications (all cells come from carcinomas): (h) enlarged centrosome, (i) string-like centrosome, (j) V-shaped centrosome, (k) sand-like centrosome, and (l) and (m) irregular shape centrosomes.

Most tumor cells showed normal γ-tubulin staining of the centrosome, with one or two rounded spots of uniform size and shape located close to the nucleus, but also a few spots near the cell membrane (Figure 1). A total of 7.3% of cells showed centrosome amplification with number changes or structural changes (Figure 2). The observed numerical centrosome amplification ranged from three to eight per cell, but most were three per cell. The structural centrosome amplification showed an enlarged centrosome (diameter of the centrosome greater than twice the diameter of the centrosome of normal control cells) with altered shape. String-like, V-shaped and sand-like irregular shapes were noted in some cells. Interestingly, in the present study we did not find any one cell that contained both numerical centrosome amplification and structural centrosome amplification.

Cells with numerical centrosome amplification were detected in all cases (75/75) of malignant lesions and in 92% (23/25) of cases of benign lesions. Cells with structural centrosome amplification were detected in 92% (69/75) of cases of breast carcinomas (two cases with ductal carcinoma *in situ *and 67 cases with invasive carcinoma), and in 12% (3/25) of cases of benign lesions (one case with mastopathias, one case with intraductal papilloma and one case with fibrocystic mastopathy).

**Figure 3:**
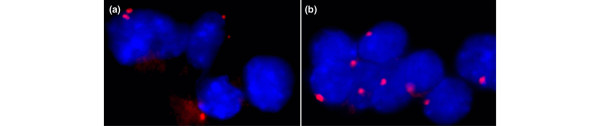
Centrosome staining of malignant tumors. Both **(a) **ductal carcinoma *in situ *and **(b) **invasive carcinoma show centrosome amplifications.

Breast carcinomas showed a mean percentage of the cells with numerical centrosome amplification of 4.86%, and a mean percentage of cells with structural centrosome amplification of 3.98% (Figure 3). These percentages were significantly higher than those in benign lesions, which presented a mean percentage of cells with numerical centrosome amplification of 2.77% (*P *= 0.000) and a mean percentage of cells with structural centrosome amplification of 0.1% (*P *= 0.000) (Table 2).

**Table 2:** Comparison of the differences in centrosome amplification between benign breast lesions and breast carcinomas

	Cases with numerical centrosome amplification	Cases with structural centrosome amplification	Cells with numerical centrosome amplification (%)	Cells with structural centrosome amplification (%)
Benign tumor	23 (92)	3 (12)	2.77 ± 2.14 (2.40)	0.10 ± 0.29 (0.00)
Malignant tumor	75 (100)	69 (92)	4.86 ± 2.66 (4.50)	3.98 ± 3.21 (3.60)
*P *value	0.10	0.00	0.00	0.00^a^

Data presented as *n *(%) or mean ± standard deviation (median). ^a^Owing to a skewed distribution, the Mann–Whitney U-test was applied.

### Cytology diagnosis and centrosome amplification

There were 91 cases that showed exact diagnostic correlation between cytology diagnoses and histology diagnoses. The other nine cases were diagnosed as suspicious carcinoma by cytology. Among these suspicious carcinomas, seven cases were diagnosed as invasive carcinomas and two cases were diagnosed as fibroadenomas by histology. All nine suspicious cases had cells with numerical centrosome amplification. Of them, six cases of invasive carcinoma also had cells with structural centrosome amplification; however, the structural centrosome amplification was not found in the cells of either of the two cases of fibroadenoma.

### Association between centrosome amplification and prognostic variables

The correlations of the centrosome amplification levels with established or proposed prognostic variables of breast carcinoma were analyzed. The mean percentage of cells with structural centrosome amplification was significantly associated with HER2/neu overexpression (*P *= 0.005) and with ER-negative status (*P *= 0.003). There was a borderline significant association between the mean percentage of cells with structural centrosome amplification and negative PR status (*P *= 0.056). The mean percentage of cells with numerical centrosome amplification was significantly associated with positive ER status (*P *= 0.03) and with positive PR status (*P *= 0.02). There were no relationships between centrosome amplification levels and other prognostic variables (Table 3).

**Table 3:** Association between centrosome amplification level and prognostic factors of breast carcinoma

Variable	Cases (*n*)	Centrosome amplification, mean ± standard deviation (%)			
		
		Structural	*P *value	Numerical	*P *value
Tumor size					
≤2 cm	40	4.01 ± 3.52	0.97	5.09 ± 3.05	0.50
>2 cm	32	3.98 ± 2.98		4.65 ± 2.25	
Nodal status					
Negative	37	4.17 ± 3.59	0.60	5.24 ± 2.99	0.26
Positive	37	3.77 ± 2.88		4.53 ± 2.31	
Histological grade					
Grade 1	12	4.03 ± 3.83		5.10 ± 1.90	
Grade 2	50	3.67 ± 3.07	0.69	5.08 ± 2.90	0.98
Grade 3	10	5.26 ± 3.60	0.11	3.65 ± 2.35	0.13
ER status					
Positive	52	3.26 ± 2.82	0.003	5.31 ± 2.72	0.03
Negative	23	5.60 ± 3.51		3.84 ± 2.30	
PR status					
Positive	63	3.6 ± 2.8	0.056	5.17 ± 2.71	0.02
Negative	12	6.2 ± 4.3		3.22 ± 1.74	
HER2/neu overexpression					
Positive	14	6.09 ± 3.97	0.005	4.01 ± 2.08	0.19
Negative	61	3.49 ± 2.84		5.05 ± 2.76	
DNA ploidy					
Diploidy	7	5.21 ± 3.81		4.07 ± 2.13	
Aneuploidy	31	4.40 ± 3.59	0.59	5.10 ± 2.86	0.36
Multiploidy	6	3.01 ± 3.62	0.40	5.37 ± 2.07	0.83

## Discussion

The present study performed centrosomal analysis using slides prepared by the ThinPrep Processor. To our knowledge, this is the first report that demonstrates the feasibility of centrosomal analysis on ThinPrep slides. Compared with the conventional slides, ThinPrep slides have a cleaner background and a more distinct cell border than conventional smears [9,10]. These features are helpful for centrosome examination.

Centrosome amplifications are found in the vast majority of human malignancies, but whether benign lesions also contain centrosome defects is still controversial. Pihan and colleagues [11] and Kronenwett and colleagues [12] found no centrosome amplification in variant normal epithelia including the breast, the prostate, the lung, the brain, the colon, and benign breast lesions. In contrast, Sato and colleagues [7] and Schneeweiss and colleagues [13] demonstrated centrosome amplifications in pancreatic adenomas, normal breast tissues and benign breast lesions. Studies in cell lines also showed that some noncancer cells contained extra centrosomes [14,15]. These cell types can apparently suppress multipolarity and form a pseudo-bipolar spindle during mitosis even though the centrosomes are amplified. Quintyne and colleagues [15] believed that clustering may be an important mechanism for preserving genomic stability in noncancer cells.

In our study, centrosome amplification – mostly numerical amplification – was found in both breast carcinomas and benign lesions. The structure of and the levels of centrosome amplification, however, were significantly different between the carcinomas and benign lesions. First, while cells with numerical centrosome amplification were found in both benign and malignant breast lesions, cells with structural centrosome amplification were mainly found in breast carcinomas: 92% of cases with breast carcinoma had cells with structural centrosome amplification, while only 12% of cases with benign lesions had cells with structural centrosome amplification. Second, the percentage of cells with numerical centrosome amplification in breast carcinomas was significantly higher than that in benign lesions. Breast carcinomas showed mean percentages of cells with numerical centrosome amplification and with structural centrosome amplification of 4.86% and 3.98%, respectively, but the mean percentages of cells with numerical centrosome amplification and with structural centrosome amplification in benign lesions were 2.77% and 0.10% (*P *= 0.000), respectively. These findings suggest that structural centrosome amplification may have a more close association with breast carcinoma. The structural centrosome amplification observed in our study, including area enlargement and shape alteration, was accompanied by an increase of the γ-tubulin immunostaining volume of the centrosome. These observations suggest that centrosomes with structural amplification may contain high levels of γ-tubulin proteins. The γ-tubulin-containing complexes are the site of microtubule nucleation, which is the key to centrosome function [16]. The increase of γ-tubulin protein may therefore be critical for centrosome function. Lingle and Salisbury [17] showed that breast carcinomas with extrapericentriolar material are more highly anaplastic than carcinomas with supernumerary centrosomes. They believed that high levels of γ-tubulin proteins in the pericentriolar material are the main cause of anaplasia.

While the structural centrosome amplification is closely related to malignance, we analyzed whether it may be an adjunct marker for FNA cytological diagnoses of breast lesions. In our study there were nine cases with cytological diagnosis of suspicious carcinoma, of which seven cases were diagnosed as invasive ductal carcinoma and two cases were diagnosed as fibroadenoma by histology. Of the seven cases with invasive ductal carcinoma, six cases had cells with structural centrosome amplification. Of the two cases of fibroadenoma, however, neither had cells with structural centrosome amplification. Centrosome detection may therefore be used as an auxiliary diagnosis for FNA cytological diagnoses. While these findings may suggest that structural centrosome amplification may be a marker, more definitive analysis with larger sample sizes will be needed.

In accordance with findings from other authors [18,19], we also detected cells with structural centrosome amplification in ductal carcinoma *in situ*, suggesting structural centrosome amplification may be an early event in the process of breast carcinogenesis.

Centrosome amplification is not only characteristic of tumors in general, but also is more pronounced in advanced-stage malignances, in recurrent tumors, and in cell lines that show more aggressive malignant phenotypes in xenograph animal models [6,20-22]. These observations suggest that centrosome amplification might be a useful marker in monitoring tumor progression. Our results demonstrated a significant correlation of the mean percentage of cells with structural centrosome amplification with HER2/neu overexpression (*P *< 0.05) and with negative ER status (*P *< 0.05), and demonstrated a borderline significant association between the mean percentage of cells with structural centrosome amplification and negative PR status (*P *= 0.056) in breast carcinomas. The mean percentage of cells with structural centrosome amplification probably predicates a more aggressive course of breast carcinoma. In line with our observation, Schneeweiss and colleagues [13] found a highly significant correlation of maximum centrosomal aberration levels with axillary nodal tumor involvement and the absence of hormone receptors in breast carcinomas. The mean percentage of cells with numerical centrosome amplification was significantly associated with positive ER status (*P *< 0.05) and with positive PR status (*P *< 0.05) in breast carcinomas. No relationship between numerical centrosome amplification with other prognostic factors of breast carcinoma was found. Additional prospective studies are again needed to confirm the observation.

In summary, the present results demonstrate that centrosome amplification including numerical amplification and structural amplification is present in breast tumors. Structural centrosome amplification has close associations with breast carcinoma and might serve as an adjunct marker in FNA diagnostic and prognostic evaluations of breast lesions.

## Abbreviations

BSA = bovine serum albumin; ER = estrogen receptor; FNA = fine-needle aspiration; PBS = phosphate-buffered saline; PR = progesterone receptor.

## Competing interests

The authors declare that they have no competing interests.

## Authors' contributions

H-qG collected samples, participated in the design of the study, read the cytology slides, and drafted the manuscript. MG and JM participated in the centrosome evaluation. TX and L-lZ carried out the immunoassays. YG participated in the design of the study. Q-jP conceived of the study, read the cytology slides, and revised the manuscript. All authors read and approved the final manuscript.
